# Medical Items

**Published:** 1893-10

**Authors:** 


### *MEDICAL ITEMS.*

---

THE next meeting of the Southern Surgical and Gynecological Association will be held in New Orleans, November 14th, 15th and 16th. The secretary, Dr. Davis, assures us that the prospects are splendid for a successful meeting. Members of the profession are cordially invited to attend.

---

WE regret to learn of the sudden death of Dr. William B. Towles, Professor of Anatomy in the University of Virginia, and in the University of Vermont. Professor Towles was one of the most thorough anatomists in this country, and possessed the art of imparting his subject in the most interesting manner. His death will be a great loss to the institutions in which he has taught with such success for many years.

---

OUR esteemed friend, Dr. J. E. Reeves, of Chattanooga, has been sued for damages by the Amick people. The doctor wrote a postal card to Dr. Mettzner, of Cincinnati, on which he stated that the many wonderful "cures" alleged to have been made by the Amick treatment in and near Chattanooga were entirely fictitious. Dr. Reeves stated that there was no evidence to show that any cases had been benefited. For all of which the Amick tribe now want damages to the extent of $20,000.

Dr. Reeves appears to welcome the fight, and says that the trial will be a Waterloo for the Amick Company. In this sort of business the doctor has a pretty good fighting record, and THE JOURNAL hopes that he may be as successful in the present instance as he has heretofore.

DR. J. L. CAMPBELL, late House-Surgeon of the Grady Hospital, has located in Atlanta, and has his office in room 330 Equitable Building, with Dr. Hutchins. Dr. Campbell will devote himself to the general practice of medicine and surgery.

---

FOLLOWING is a lay comment taken from a late issue of the Boston *Herald*. Massachusetts and Georgia are "in the same boat," and the remarks here quoted are as applicable to the latter as to the former:

Connecticut is now added to the list of States where the practice of medicine is regulated by law, the bill relating to this subject having passed the legislature of that State. There are now but nine States in the Union where the practice of this profession is absolutely unrestricted by any rules whatever, and strange to say, Massachusetts is one of the delinquent States. The only equipment that is essential for the practice of medicine in Massachusetts is a sign board hung outside the physician's office, and even this is frequently dispensed with. Massachusetts is the practitioners' paradise.

---

WE learn with regret of the death of Dr. Graily Hewitt, the distinguished Professor of Obstetrics and Diseases of Women in the University Medical College, London. Dr. Hewitt had made many important contributions to the literature of his special branch of medicine and surgery, and he enjoyed in a peculiar degree a wide personal popularity. His principal work was his "Diseases of Women," which was for many years one of the most popular text-books on this subject. Among other valuable contributions to the literature of obstetric medicine may be mentioned "Uncontrollable Vomiting of Pregnancy," "Clinical Notes on Sterility," "Nutrition the Basis of Treatment in Disease," and several papers on Displacements and Flexions of the Uterus.

THOSE who have had the pleasure of reading that interesting autobiography, "A Doctor's Experience in Three Continents," will regret to learn of the death of the distinguished author, Dr. Edward Warren Bey. Dr. Warren was a native of North Carolina where he practiced medicine for some years. He was surgeon in the Confederate army and after the war became professor in one of the medical colleges in Baltimore. Later he accepted a commission in the Egyptian army and rapidly rose to the rank of surgeon-general. While on a visit to Paris he determined to locate there. For the past several years he has been one of the most prominent of the practitioners of Paris, being especially popular with the American colony.

---

WE are indebted to our friend, Dr. C. W. Tompkins, of Jasper, Fla., for the following poetic clipping from a late issue of the Jasper *News*. Dr. Tompkins has had it printed on the back of a number of his statements:

Once upon a midnight dreary,
The doctor slumbered weak and weary,
And all the town could hear
Him snore.

While he lay there sweetly napping,
Suddenly there came a tapping
Like a ramgoat madly rapping
His hard head upon
The door.

"Get thee up" a voice said loudly,
"Come in haste" it added proudly,
Like a man who owned a million or
Much more.

But the doctor never heeded,
Back to dreamland fast he speeded,
For such men as that he needed
In his practice
Nevermore.

For long months that man had owed him,
Not a cent he'd ever paid him,
And the doctor now will dose him
Nevermore.

### TRI-STATE MEDICAL SOCIETY OF ALABAMA, GEORGIA AND TENNESSEE.

The Fifth Annual Meeting of the Tri-State Medical Society will be held in the Unitarian Church, Chattanooga, October 17th, 18th and 19th, 1893. The indications point to a large and interesting meeting. Those desiring to present papers should send title of same to the secretary without delay. The following is a partial list of papers to be read :

"Membranous Croup, with Report of Cases. Treated with Tracheotomy." R. B. Harbin, Calhoun, Ga.

"Treatment of Stone in the Cystic Duct." W. E. B. Davis, Birmingham, Ala.

"Choleocystotomy." Paul F. Eve, Nashville, Tenn.

"Symptoms and Pathology of Fractures about the Elbow." J. B. Murfree, Murfreesboro, Tenn.

"Treatment and Prognosis of Fractures about the Elbow." Willis F. Westmoreland, Atlanta, Ga.

"Pathology of the Sequelæ of Purulent Inflammation of the Middle Ear." T. Hilliard Wood, Nashville, Tenn.

"Treatment of the Sequelæ of Inflammation of the Middle Ear." G. C. Savage, Nashville, Tenn.

"Action of the Galvanic Current on the Uterine Tissues." H. Berlin, Chattanooga, Tenn.

"The Significance of Albumin in the Urine in Pregnancy." E. T. Camp, Gadsden, Ala.

"Tuberculosis on the Cumberland Mountains." L. P. Barbour, Tracy City, Tenn.

"Symptoms and Treatment of Gastritis." G. T. Prince, Whiteside, Tenn.

"Treatment of Varicocele.' J. W. Handly, Nashville, Tenn.

"Treatment of Pyrexia." J. A. Witherspoon, Columbia, Tenn.

"Fevers of Alabama." R. M. Cunningham, Birmingham, Ala.

Papers are also expected from J. B. Cowan, Tullahoma, Tenn.; G. I. Cullen, Cincinnati, Ohio; F. W. McRae, Atlanta, Ga.; J. P. Stewart, Attala, Ala.; Max Thorner, Cincinnati, Ohio; M. B. Hutchins and L. B. Grandy, Atlanta, Ga., and others.

An invitation is extended to all who desire to attend the sessions of the coming meeting. FRANK TESTER SMITH, Sec'y,
Chattanooga, Tenn.

